# Nanoparticle-Mediated Therapeutic Agent Delivery for Treating Metastatic Breast Cancer—Challenges and Opportunities

**DOI:** 10.3390/nano8060361

**Published:** 2018-05-24

**Authors:** Yunfei Li, Brock Humphries, Chengfeng Yang, Zhishan Wang

**Affiliations:** 1 Department of Toxicology and Cancer Biology, and Center for Research on Environment Disease, College of Medicine, University of Kentucky, Lexington, KY 40536, USA; 2 Department of Pharmaceutics, Institute of Medicinal Biotechnology, Peking Union Medical College, Beijing 100050, China; 3 Department of Radiology, University of Michigan, Ann Arbor, MI 48109, USA

**Keywords:** breast cancer, metastasis, nanomaterials, nanomedicine

## Abstract

Breast cancer (BC) is the second leading cause of cancer-related death in American women and more than 90% of BC-related death is caused by metastatic BC (MBC). This review stresses the limited success of traditional therapies as well as the use of nanomedicine for treating MBC. Understanding the biological barriers of MBC that nanoparticle in vivo trafficking must overcome could provide valuable new insights for translating nanomedicine from the bench side to the bedside. A view about nanomedicine applied in BC therapy has been summarized with their present status, which is gaining attention in the clinically-applied landscape. The progressions of drug/gene delivery systems, especially the status of their preclinical or clinical trials, are also discussed. Here we highlight that the treatment of metastasis, in addition to the extensively described inhibition of primary tumor growth, is an indispensable requirement for nanomedicine. Along with more innovations in material chemistry and more progressions in biology, nanomedicine will constantly supply more exciting new approaches for targeted drug/gene delivery against MBC.

## 1. Introduction

Breast cancer (BC) is the second leading cause of cancer-related death in women. Over 252,710 new cases of invasive BC were diagnosed and 40,610 BC deaths were expected to occur in 2017 in the United States alone [1]. Recently, because of rapid progression in imaging, surgery, radiation, chemotherapy, and endocrine therapy, the mortality rate of BC patients has decreased [1]. However, even for patients initially diagnosed with early stage disease, 20–30% will ultimately develop metastatic disease [2]. Furthermore, more than 90% of BC-related mortality is associated with metastasis [3], which highlights the urgent need to identify and implement more effective BC therapies. Recent advances in genomics and cancer biology have demonstrated notable heterogeneity between BC subtypes [4,5,6]. This heterogeneity can greatly contribute to metastasis, which poses a significant challenge for designing effective BC treatments. Migration is one of the key features that a cancer cell must obtain to successfully metastasize, and cells can gain motility through activation of the epithelial-to-mesenchymal transition (EMT) program. EMT is typically characterized by cytoskeletal rearrangement as well as morphological changes and is thought of as a major contributor to the metastasis of epithelial-originated BC. In addition to enhanced motility, EMT has been shown to contribute to metastasis by increasing invasion and apoptosis resistance [7]. Therefore, to develop successful treatments against metastatic breast cancer (MBC), these barriers must be overcome.

Nanomedicine is an attractive field of translational medicine that can be used against metastatic cancer. Various forms of tumor/metastases-targeting nanomedicine have been attempted to address this need and this field is expected to drive therapeutic research forward [8,9,10,11,12,13,14,15,16,17,18,19,20,21]. Nanomedicine offers numerous benefits over other options, which include (i) targeted delivery to the desired sites, (ii) the capability to load single or more therapeutic agents and, (iii) the ability to overcome solubility and stability issues. Doxil^®^ was the first Food and Drug Administration (FDA) approved nanomedicine against metastatic ovarian cancer and AIDS-related Kaposi’s sarcoma in 1995, which was formulated to improve the efficacy and reduce the toxicity of doxorubicin therapy. In 2005, Abraxane^®^ was approved to treat MBC and has been shown to improve tumor uptake of paclitaxel [22]. Abraxane^®^ is a typical albumin-bound nanoparticle with the size of 130 nm designed to avoid Cremophor^®^ toxicities and delivers paclitaxel through albumin-binding proteins [10,11,12]. Nanomedicine is also used in the diagnosis of cancer metastasis. Cornell Dots^®^, currently in clinical trials, are silica spheres labeled with radioactive iodine for PET scans, which could be used to determine metastatic sites by identifying the location of Cornell Dots^®^ accumulation [23]. Therefore, there is a strong potential for the expansion of nanomedicine in to metastasis treatments to improve patient outcome.

Concurrent with the progress of nanomedicine in cancer, non-coding RNAs (ncRNAs) have also shown great potential in treating MBC. NcRNAs are RNA molecules that do not have protein coding capacity. Compared to conventional chemotherapeutic antineoplastic drugs, ncRNAs as therapeutics can offer many advantages. Thousands of ncRNAs with unique sequences have been identified within cancer cells and have been experimentally associated with cancer compared to normal tissue. In every major cancer type, there have been reports of ncRNAs eliciting tumor suppressor or oncogenic inducer effects [24]. The aim of ncRNA interference-based therapy is to reduce the expression of ‘uncontrollable’ genes involved in cancer, especially against undruggable targets. Over the past two decades, while many ncRNAs have been successfully used in experimental models, their clinical applications are limited due to lack of proper delivery systems for ncRNAs. Therefore, developing nanomaterial-based ncRNA delivery platforms would provide additional powerful tools for the potential treatment of various diseases including MBC.

This review will focus mainly on drug/gene including ncRNA gene delivery nanoparticles and not on nanoparticle contrast agents for medical imaging. This review will cover the milestone advances within this field and current clinical trials, as well as challenges and opportunities for nanomedicine in the treatment of cancer progression and the prevention of metastasis. As a new era of personalized cancer medicine is quickly developing, nano-delivery platforms provide more options for the modular personalized therapies. These nano-delivery platforms in conjunction with studies of the biological mechanisms underlying MBC metastasis will help facilitate the design of individualized strategies for patients, and thus elongate overall survival duration.

## 2. Traditional Cytotoxic Chemotherapy and Targeted Therapy against MBC

Analysis of the gene expression profiles and the immunohistochemical expression of estrogen receptor (ER), progesterone receptor (PR) and human epidermal growth factor receptor 2 (HER2), has been used to divide breast carcinomas into five intrinsic subtypes: luminal A, luminal B, HER2-enriched, basal and claudin-low [4]. Currently, the treatment of BC is based upon several different factors, including BC stage and progression. The stage is mainly determined by tumor size, number and location of lymph nodes being invaded, and the presence/absence of distant metastatic foci [25]. Therapeutic strategies include surgical tumor resection, broad-spectrum and targeted therapy. What makes BC difficult to treat is that each subtype has a different response to the therapeutic strategies, thus affecting prognosis. The luminal A, luminal B, and HER2 subtypes are sensitive to targeted hormone therapy since ER and HER2, respectively, are therapeutic targets. Although endocrine therapy utilizing tamoxifen and raloxifene (for ER) is effective in treating luminal A and luminal B subtypes of BC [25,26], HER2-enriched BCs are not responsive and thus HER2-targeted therapy using trastuzumab is developed for tamoxifen-resistant HER2-enriched BC [27]. However, unlike luminal and HER2 subtypes, basal and claudin-low subtypes, which consist of most MBCs, are more biologically aggressive and particularly difficult to treat. Since being characterized by the lack of expression of ER, PR and HER2, basal and claudin-low subtypes are sometimes defined as triple-negative BC (TNBC). Compared to non-TNBC patients, TNBC patients usually have a poor prognosis [28]. Particularly, TNBC accounts for 15–20% of all BCs and displays higher risk of relapse and metastasis. However due to the lack of ER, PR and HER2 expression, both endocrine and HER2-targeted therapeutic strategies do not show tumor response in TNBC patients [26,29]. Therefore, chemotherapy is currently the only option of systemic administration therapy for TNBC patients [30].

MBC signaling is complex. As a result, response to the treatment of MBC with a single drug is limited and most of response rates of single-used chemotherapies on MBC patients are under 60% [25]. Moreover, the inhibition of a single pathway usually causes a high incidence of drug resistance and tumor relapse. Although various chemotherapies can reduce tumor size [25], measurable reductions do not necessarily correlate with an improvement in progression free or overall survival (OS) for patients [25]. Focusing on TNBC, a study exploring the relationship between breast-cancer subtypes and response to chemotherapy demonstrated that TNBC patients had significantly poorer rates of progression-free survival (*p* = 0.04) and OS (*p* = 0.02), compared with the other BC subtypes [31]. This study concluded that although TNBC is responsive to chemotherapy, it is more resistant to standard chemotherapeutics than other subtypes of breast cancer and therefore these patients have a poorer prognosis. Over the years, combination therapy has gradually become the dominant chemotherapy treatment method in the clinic, which could overcome multi-drug resistance and improve patient response rate through modulation of diverse signaling pathways (synergistic effect) [32]. Furthermore, combination therapy could improve drug efficiency at a lower administrating dosage, and hence reduce individual drug-associated toxicity [32]. For example, the combination of methotrexate and mitomycin C has demonstrated potent suppressing effect in MBC patients pretreated with taxanes or anthracyclines [33]. However, due to the different pharmacokinetic profiles of combinational drugs in vivo, the synergistic effects are largely compromised compared to in vitro studies and conclusions are not as overwhelming as we expected.

Currently, effective strategies for treating MBC are severely lacking. Molecular-targeted therapies are the gold standard for MBC treatment, and, to a certain extent, targeted therapies have marked a new era of MBC treatment. The targets of these drugs mainly include serine-threonine kinases, growth factor receptors and non-receptor signaling molecules [34]. For instance, the first clinically-applied targeted therapy was Herceptin, a monoclonal antibody against receptor tyrosine kinase HER2 and approved by the FDA for treating patients with HER2-positive BC in 1998 [35]. However, a single targeted therapy is far from efficient to conquer all kinds of cancers due to inter-tumoral heterogeneity and resistance resulting from the heterogeneity of metastatic foci. For example, it has been shown that although chemotherapy can significantly increase 15-year survival rate of patients suffering from early stage BC, BC recurrence and metastasis still occurred in a large proportion of these patients [36]. Hence, sufficient delivery of drug to metastatic sites remains a critical barrier to sustain the benefits of precision medicine in MBC therapy.

## 3. Current Techniques Used for ncRNA Delivery in Cancer Therapy

**Figure 1:**
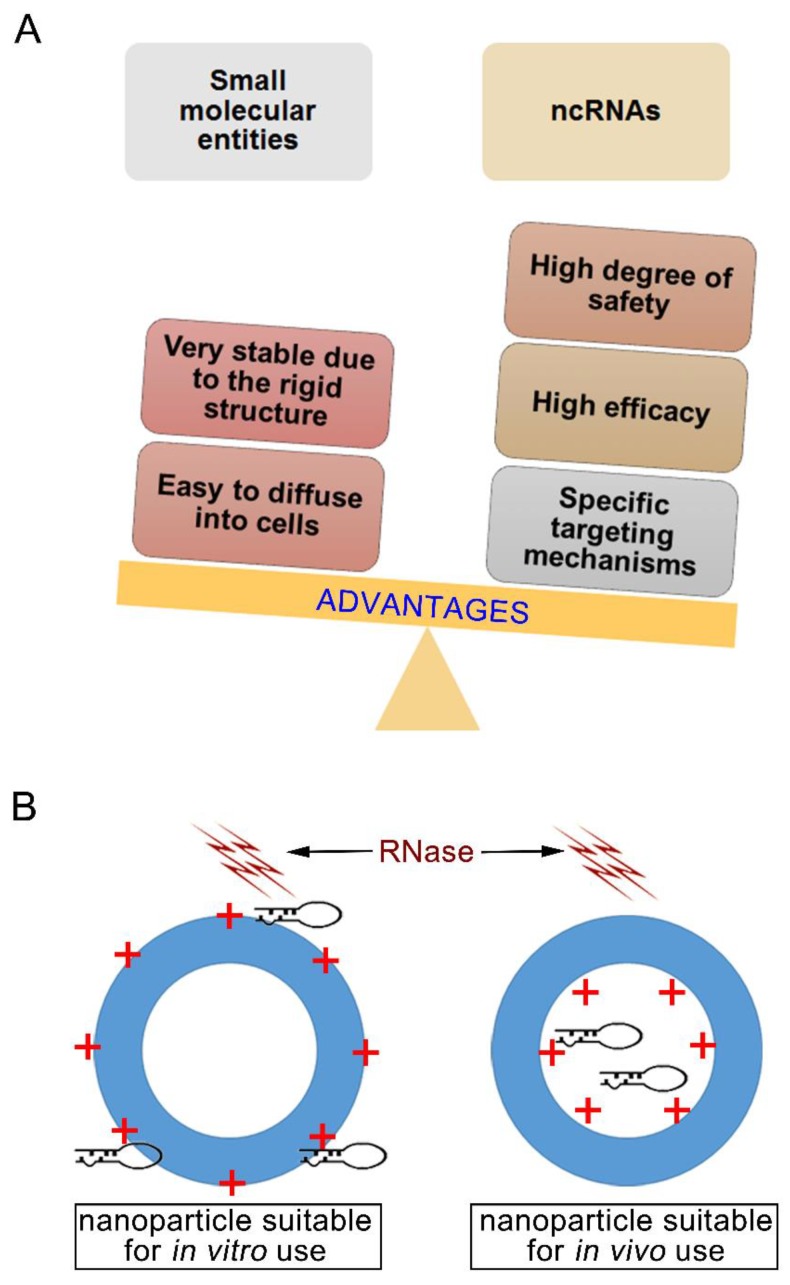
The advantages of ncRNAs against metastatic breast cancer (MBC) and the general structures for the non-viral delivery vehicles. (**A**) The advantages comparison between small molecular entities and ncRNAs. (**B**) The nanostructure difference between in vitro and in vivo applied nanoparticles. Aiming to prevent the degradation by RNase in vivo, ncRNAs must be loaded into the core of nanoparticles. Loading into the core of the nanoparticle will allow the ncRNA to be successfully applied in clinical settings.

Due to their unique mechanisms, ncRNAs have three advantages as a potential cancer therapeutic strategy over cytotoxic chemotherapy (Figure 1A): (1) they have a high degree of safety. NcRNAs such as microRNAs (miRNAs) elicit their roles at the post-transcriptional level of gene expression without direct interaction with genomic DNA, so the risks of mutation and teratogenicity resulting from miRNA gene therapy are significantly reduced. (2) they have high efficacy, and (3) they have advantageous targeting mechanisms. MiRNAs have a large number of potential targets and high specificity due to the principle of complementary base pairing when compared to the other small molecular entities. However, these advantages are not exclusive to miRNAs, as gene silencing effects can be achieved by other ncRNAs such as small interfering RNAs (siRNAs) and double-stranded hairpin RNAs (shRNAs).

However, to become clinically relevant ncRNAs must overcome the following barriers: (1) naked ncRNAs are quite unstable in human plasma, and (2) they are too negatively charged and large to cross the cellular membranes. Aiming to avoid the instability issue, the currently used ncRNAs are mainly produced by chemical synthesis and are artificially modified [37]. However, it has been reported that these chemical modifications of ncRNAs may induce immunogenicity through toll-like receptors [38,39]. Furthermore, in vitro/in vivo delivery of chemically modified siRNAs without of nano-delivery vehicles remains a big challenge and has been reviewed elsewhere [40]. Delivery by viral-vehicles is another way to efficiently introduce ncRNAs to cells, but it is highly restricted by the safety concerns arising from intractable virions. Moreover, inherent immunogenicity is another big issue with viral delivery vehicles, which could result in severe immune reactions and impede further administrations. The limited targeting capability and high cost are also problems that a viral delivery vehicle must address. However, by taking advantage of rapidly-advancing nanotechnology, the development of non-viral delivery vehicles containing ncRNAs could achieve their biological effects without triggering antigenicity.

Currently, siRNAs have quickly crept into biomedical research as a new powerful tool for the potential treatment of various diseases, and several promising clinical studies using nanotechnology have reported success (Table 1). Therefore, the use of siRNA-based therapy is one possible therapeutic avenue that can have success against MBC from a pharmaceutical development perspective.

Outside of siRNAs, miRNAs represent another family of endogenous small ncRNAs which have attracted much attention because of their ability to modulate the expression levels of target proteins involved in almost all physiological functions. More than 2600 miRNAs have been reported in human cells [41], and some of them have been investigated as potential MBC therapeutic agents (e.g., *let-7a* targeting HER2/Aurora-B pathway [42], *miR-21* targeting E-cadherin-ZEB1/2 pathway [43], *miR-27a* targeting MET, EGFR, and PI3K-AKT pathway [44], *miR-34a* targeting AXL [45], *miR-155* targeting VHL, TP53INP1, and PI3K-AKT pathway [44,46,47], *miR-181* targeting K-Ras [48], *miR-200b* targeting PKCα [49], *miR-221* targeting PTEN, p27kip1, p57kip2, and PUMA [50], *miR-301a* targeting PTEN [51]).

With current research focusing on elucidating the mechanisms and targets of siRNA and miRNA, these provide an area of research that can deliver breakthroughs for the treatment of MBC metastasis. However, there is still a gap in knowledge that nanotechnology can help fill. The major hurdles including the instability in the blood and inability to pass through the cell membrane prevent siRNAs and miRNAs from clinical use. Below we describe the two main delivery vehicles for siRNA and miRNA, which have the general schematic as outlined in Figure 1B.

### 3.1. Lipid-Based Vectors for Antineoplastic Gene Delivery

Lipofectamine 2000 (lipo2000) is an extensively-used cationic lipid formulation for in vitro siRNA transfection. Lipo2000 and another newly developed derivative, Lipofectamine RNAimax, can improve the transfection efficiency by up to a 1000-fold [52]. Despite being effective as siRNA transfection agents in vitro, they are toxic, which restricts its applications in therapy settings.

Because the surface charge is negative, neutral liposomes are usually more biocompatible and have better pharmacokinetics than cationic liposomes. 1,2-dioleoyl-sn-glycero-3-phosphatidylcholine (DOPC) is an example of a neutral lipid and is used extensively to entrap siRNA. As shown in Table 1, the EphA2 targeting DOPC-encapsulated siRNA liposome is currently in a phase 1 clinical trial for the treatment in patients with advanced and/or recurrent solid tumors.

However, neutral liposomes have repulsive forces with cell membranes and are not easily endocytosed by cells, so cationic liposomes are still preferred for transfections. Superior cationic liposomes, such as Lipofectamine RNAimax, have also been designed to overcome the limitations of cationic liposomes. In addition to Lipofectamine RNAimax, other superior cationic liposomes have been designed and show promise for disease treatment. For instance, the most well-known cationic lipid-based vector is the SNALP (stable nucleic acid lipid particle), which consists of dioleoyl-phosphatidylethanol-amine and 1,2-dioleoyl-3-trimethylammonium-propane (DOTAP), and is designed for in vivo delivery of therapeutic siRNAs in mammals. DOTAP is a superior cationic lipid which has been demonstrated to form cationic liposomes with negatively charged siRNA [53]. Extensive studies regarding the use of DOTAP-based SNALPs in disease have been reported [16,17,18,19,20,21]. Furthermore, as shown in Table 1, Tekmira Pharmaceuticals initiated a phase 1 clinical trial of a SNALP-encapsulated siRNA targeting Plk1 (TKM080301) in adult cancer patients in 2010, and Alnylam Pharmaceuticals has initiated a phase 1 clinical trial of the first dual-targeted siRNA drug SNALP-delivered siRNAs targeting KSP and vascular endothelial growth factor (ALN-VSP02) for the treatment of advanced solid tumors in 2009, demonstrating that SNALPs are likely useful for drug delivery.

### 3.2. Polymer-Mediated Gene Delivery Systems

Polymer-based delivery systems are usually called polymeric nanoparticles and have been used extensively as drug nanocarriers. Cyclodextrin (CD) is one of the most reported polymers for gene delivery, and the CD-based delivery system was first reported for the delivery of plasmid DNA in 1999. Soon thereafter CD-mediated nanoparticles advanced into clinical trials, which was the first targeted siRNA delivery system entering clinical trials for cancer treatment [8]. In this CD-based delivery system, both adamantane(AD)-PEG and AD-PEG-transferrin were used to improve delivery efficacy in vivo, a process known as PEGylation. This is because AD can be stably included into the CD core and the PEG corona can protect the nanoparticle from blood clearance by reducing the interaction with serum proteins. However, these conjugates also decrease cellular uptake and silencing efficacy, citing an important caveat of this system [54]. Using this system as a benchmark, Calando Pharmaceuticals have developed CALLA-01, which targets riboneucleotide reductase to inhibit tumor growth [55].

Although PEGylation of a nanocarrier can avoid renal clearance and phagocytosis, it should be noted that PEGylation could also hinder the cellular interaction by a phenomenon called the ‘PEG dilemma’. Various strategies have been implemented to avoid this obstacle. We developed a PEGylated nanoparticle (NP) using a complex coacervation of PEI/PEG-P(asp), and successfully transfected reporter genes in vivo [13]. Despite being hidden under the PEG corona, complex coacervation can still dominate the surface charge to be positive to facilitate the interaction with cells. Once endocytosed, PEI in the complex coacervation could be triggered to release the cargo via the sponge effect.

Although steps have been taken to fill in the gaps of knowledge, more research is needed to translate therapeutic non-coding RNAs into clinical reality. For example, several clinically tested siRNA therapeutics are administrated intravenously by synthetic carriers, and none of them have been reported to be routinely used in the clinic. Many of the unsuccessful nanomedicine-based clinical trials cited poor efficacy, inability to achieve primary objectives, or have safety problems. Therefore, there is an urgent need to develop more concrete delivery systems.

## 4. The Clinically Applied Drug-Based Nanomedicine against Cancer and Its Confronted Challenges

The inefficiency of traditional therapies provides a unique opportunity for nanomedicine (Abraxane^®^) to intervene for treatment of MBC. Nanomedicine is a cutting-edge interdisciplinary field that has developed rapidly and widely over the past 20 years. Originating from biology, organic and inorganic chemistry, nanocarriers have helped medicine to treat various cancers through multiple biological mechanisms and targets (Table 2). Lipid-based and polymeric materials constitute the majority of the nanocarriers because of their unique properties that enable the anchorage of targeting moieties, degrade under physiological conditions, and carry a large amount of drug molecules.

Advances in nanotechnology greatly contribute to our ability to treat MBC. One such example of nanomedicine applied in BC treatment is Abraxane^®^. Paclitaxel has poor water solubility and hence usually formulates with a large amount of polyoxyethylated castor oil (Cremophor^®^). This formulation is favorable for drug delivery because of its favorable safety profile and greater therapeutic index compared with standard paclitaxel (Cremophor^®^-based) in patients. However, Cremophor^®^ is not an inert excipient and has been shown to demonstrate unwanted reactions. Therefore, Abraxane^®^ was developed to avoid these complications while improving drug delivery. In a phase 3 clinical trial against MBC, Abraxane^®^ was administered at 260 mg/m^2^ every 3 weeks (q3w) while standard paclitaxel was administered at 175 mg/m^2^ q3w [10]. This study demonstrated that Abraxane^®^ had a much lower incidence of grade 4 neutropenia, a significantly superior overall response rate (RR) and a significantly longer time to progression (TTP). However, it was also found that Abraxane^®^ did not show any significant improvement on patient OS, suggesting this nanomedicine has a limited success in this trial. One possibility for the inability of Abraxane^®^ to improve OS is due to the inadequate targeting capability of the particle. Additionally, there have been 7 albumin-binding proteins discovered, a large portion of which are located at the normal cells apart from tumor cells [56], therefore the drug could have bound to any of these proteins instead of its intended target. However, more research will need to be done to identify the potential uses for Abraxane^®^.

In addition to paclitaxel in Cremophor^®^, Abraxane^®^ has also been compared to docetaxel for the treatment of MBC in clinical trials. In this study, Abraxane^®^ administered by three distinct dosing regimens (300 mg/m^2^ intravenous q3w, 150 and 100 mg/m^2^ intravenous the first 3 of 4 weeks (qw 3/4)) was compared to docetaxel (100 mg/m^2^ intravenous q3w). RRs were similar between Abraxane^®^ and docetaxel given q3w (33% vs. 36%) [11,12]. However, the 150 mg/m^2^ qw 3/4 dose of Abraxane^®^ was found to be a significantly more effective regimen than docetaxel [12], and median OS of 150 mg/m^2^ qw3/4 was 33.8 months compared with 26.6 months for docetaxel [12].

Although the efficacy of nanotherapeutics is improving, it is still a major challenge for engineers to consider the limited therapeutic efficiency against MBC metastasis. Increasing evidence suggests that the regulation of metastasis differs greatly from primary tumor inhibition, and therefore casts doubt on the clinical validity of using traditional nanomedicine. For example, the large particle sizes can hinder the delivery of Abraxane^®^ and Doxil^®^ deep into the tumor parenchyma, which resulted in only a modest survival benefit to patients [10,57,58,59]. Furthermore, single use nanomedicines were also found to be inefficient in treating MBC metastasis [14,60]. One of the major challenges that face nanomedicine remains the ability to target the metastatic lesions within a large population of normal cells, which are substantially distinct from the primary tumor. For example, traditional nanotherapeutics methods that are effective for treating primary tumors via Enhanced Permeation and Retention (EPR) effect may be inadequate while dealing with small clusters of disseminated malignant cells. Another major challenge is the limited in vivo stability of nanoparticles. However, many nanoparticles reportedly claim their excellent stability via demonstrating the extended in vitro release profile, and most of them will dissociate when suffering from shearing forces in the circulation system and the premature-released payloads must arrive at the desired sites in a low efficiency without any passive or active targeting effects. It has been reviewed elsewhere that Abraxane^®^ did not show significantly improved pharmacokinetics and biodistribution due to the rapid dissociation upon intravenous injection and thus contribute little to OS [61]. Prior to talking about the targeting effect from nanomedicine, more advances are still needed to provide more concrete groundwork for developing nanoparticles with better in vivo reliabilities. Hence, a better understanding of the fundamental processes involved, which include nanoparticle circulation, biodistribution, tumor/metastases targeting, and tumor/metastases penetration, is desperately needed to overcome the major hurdles in nanoparticle-based therapy.

## 5. Biological Challenges Associated with Treating MBC

To achieve the therapeutic goal, it is important to understand the complexity and interplay between the biology of MBC metastasis and the fabricated nano-system.

### 5.1. Challenge 1—Limited Access to the Dormant Cells or Micrometastases

**Figure 2:**
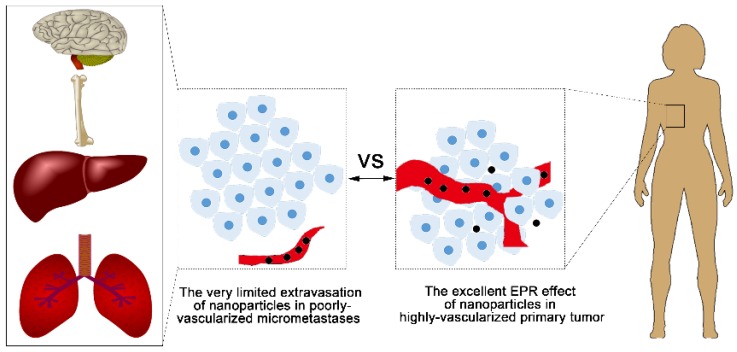
The limited Enhanced Permeation and Retention (EPR) effect in micrometastases. Nanoparticles circulating in the blood could accumulate in a large and well-vascularized primary tumor via EPR effects; in contrast, micrometastases are poorly vascularized and restrict the access of nanoparticles.

Compared to the eradication of primary tumors, identification and delivery of therapeutic agents to metastatic lesions is a much bigger challenge. Once the cancer cells are disseminated into a secondary organ, there are three distinct kinds of niches that coexist within the metastatic lesion: single dormant cells, dormant micrometastases, and actively growing and vascularized metastases, which may be detected by their effects on vital organ function or by various imaging instruments [62]. To date, few studies have investigated the effects of drugs on dormant cancer cells. In models of BC metastases to the liver, cytotoxic chemotherapy effectively inhibited the development of metastases, but had little effect on the elimination of dormant cancer cells disseminated to liver [63]. Furthermore, another study demonstrated that chemotherapy treatment given at the early stage of metastasis had no effect on late-developing BC metastases, presumably due to a dormant state of the cancer cells at the time of treatment [63]. Therefore, dormant cancer cells remain an elusive target for therapy using small-molecular drugs and provide a greater challenge for nanomedicine which uses a much larger size to deliver therapeutic agents. When used to target micrometastases and poorly-vascularized metastases, nanotherapeutics also encounter the same problem. The primary tumor vasculature can have fenestrae up to 600 nm and tumor-associated lymphatic drainage is often poor, therefore, some solid tumors will exhibit an EPR effect for nano-vehicles [64]. However, the EPR effect is only present in tumors of more than ~100 mm^3^ in volume, and fails in the unvascularized metastases [65]. This greatly hinders the application of nanotherapeutics in the treatment of MBC metastasis. Similarly, the strategy of targeted delivery has been well established for primary tumors, but it only has a few applications in the treatment of metastatic foci (Figure 2). The targeting method may be adapted to metastases by addressing unique site tags on the populations of cells that form metastatic foci or by exploiting the metastatic phenotype, but identifying these unique tags is still in its infancy. Generally, the smaller size, dispersion in the body, and the presence of less vasculature than primary tumors are unique physiological barriers of metastases, which make metastases less accessible to molecular and nanoparticle agents [66].

### 5.2. Challenge 2—The Anti-Apoptosis Characteristic of Cancer Stem Cells (CSCs)

In general, undifferentiated CSCs seem to coexist with the fully differentiated end stage cells and partially differentiated transit amplifying cells simultaneously in breast carcinoma [67]. One feature that helps to define a CSC is its unique ability to resist apoptosis [3]. Metastasis is a truly inefficient process-despite of large number of cells migrating away from primary tumor, only a few successfully colonize a secondary organ. One proposed reason is provided by the CSC model, which hypothesizes that colonization can only be achieved by CSCs, which are rare within BC primary tumors [68,69]. As few as 100 to 200 breast CSCs have been shown to develop tumors when being inoculated into the mammary fat pad of immunocompromised mice [70]. Furthermore, CSCs are one of the major causes of treatment resistance in MBC patients. This resistance presumably stems from two sources: (1) CSCs often retreat from the activated G1/S phase into G0 phase; and (2) the close association between CSCs and the mesenchymal cancer cells. Mesenchymal cells have been shown to be the product of EMT and typically exhibit intrinsic drug resistance [71]. In support of this, various studies have identified that the drug-resistant cancer cell subtype often exhibit a more mesenchymal-like phenotype [72].

## 6. Designing Nanomedicine Strategies to Overcome the Limitations in MBC Metastasis

**Figure 3:**
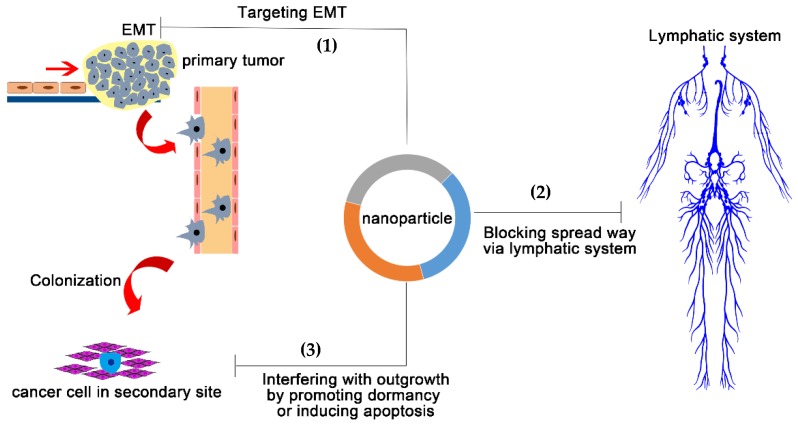
Three potent strategies to stop MBC metastasis via nanotechnology. (1) Reversing Epithelial Mesenchymal Transition (EMT) to reduce the mobility of MBC cells in the primary tumor site. (2) Using nanoparticles to stop cells from spreading through the lymphatic system (such as ultra-small nanoparticles with the diameter < 30 nm) or activate the lymphatic system to capture MBC cells in transit. (3) Enforcing the dormancy of already localized MBC cells or inducing their apoptosis.

Metastasis is a particularly complex process, and metastatic dissemination constitutes the following steps including: invasion of surrounding tissues, entry into the microvasculature of the lymphatic and blood systems, transportation by the circulatory system, arrest and survival in the microenvironment of secondary organs, and finally, colonization [73]. From a treatment landscape, understanding the underlying mechanisms of the early steps is important for the treatment of early-stage MBC patients, whereas understanding the mechanisms of successful colonization is very important for the effective therapies for patients with already-established metastasis [3]. Accordingly, the complex metastatic cascade can be simplified into two major steps: (1) physical translocation of the cancer cells away from the primary tumor to secondary organs and (2) colonization of distant organs. The distinct capabilities of nanoparticles in targeting, detection and trafficking in vivo [74], are expected to enable novel approaches to effectively target both of these steps. However, the genetic and biochemical determinants of CSC colonization at the secondary site are unclear, which renders more difficulties to the rational design of nanomedicine. As the biological mechanisms of tumor cell dissemination are disclosed, feasible nanotherapeutics approaches to treat MBC may be designed accordingly (Figure 3).

### 6.1. Preventing the Physical Translocation Away from Primary Tumor to Secondary Organs

#### 6.1.1. Targeting Epithelial-to-Mesenchymal Transition (EMT) of Primary Tumor

Targeting the primary tumor with anti-migratory ncRNAs to keep cells in place is currently very effective in preclinical studies. During the EMT process, normally polar, epithelial breast cells switch to a more motile and invasive mesenchymal-like phenotype. Certain families of miRNAs have been found to potently regulate EMT. For instance, the *miR-200* family, known for directly targeting the E-cadherin suppressors zinc finger E-box binding homeobox 1 and homeobox 2 (ZEB1 and ZEB2), was found as a promising family against cancer metastasis [75,76]. More specifically, the *miR-200* family member, *miR-200b*, prevents TNBC cell migration and metastasis by suppressing Protein Kinase Cα (PKCα), which is a member of PKC family of serine/threonine kinase constituted by 10 isozymes, crucial in regulating cell migration and TNBC metastasis [49].

Furthermore, other miRNAs have also been shown to have potential therapeutic effects against EMT clinically. *miR-34a* was found to be significantly downregulated in TNBC cell lines. One of the direct targets of *miR-34a* is AXL, which is a member of the TAM (TYRO3-AXL-MER) receptor tyrosine kinase family and plays a diverse role in survival, proliferation, migration, invasion and angiogenesis [45]. High AXL expression level correlates with poorer prognosis of MBC patients [77], and therefore has emerged as a promising target for cancer therapy. Various AXL inhibitors have been developed, but only one has been found to be efficacious against MBC metastasis in a preclinical study [78]. A nanoparticle-mediated *miR-34a* mimic (MRX34) was reportedly developed, and has become the first microRNA-based therapy to reach phase 1 clinical trials in 2013 [15]. However, this study was terminated by FDA in 2016 because of immune-related severe adverse events, likely caused by the applied delivery vehicles. This further emphasizes the importance of studying and elucidating the mechanisms of an effective systemic delivery system, including viral and non-viral vehicles. Currently, the non-viral vehicles mainly consist of nanoparticle-based delivery systems. Compared to systems delivering other kinds of gene therapeutics, the delivery of miRNAs is especially challenging because of the natural characteristics of miRNAs. As an emerging delivery mechanism, nanotechnology is a valuable tool for clinical application of miRNA-based therapies.

Since EMT is closely correlated with chemo-resistance [79], the design of a drug/gene codelivery system to attenuate CSCs and EMT is a potential avenue to avoid drug resistance and relapse of MBC. Hammond and colleagues, have developed a multi-layered nanoparticle for systemic codelivery of siRNA and doxorubicin for MBC treatment [80]. The advantage of this codelivery system is that the attenuation of the corresponding gene(s) could create a transient time window in which the resistant MBC cells become vulnerable to the antineoplastic drug, thereby overcoming multidrug resistance resulting from MBC. Furthermore, development of intelligent gelatinase-sensitive nanoparticles co-delivering docetaxel and *miR-200c* was also able to inhibit EMT and showed promise for cancer therapy [81]. Both examples provided very good reference for MBC treatment.

#### 6.1.2. Blocking the Spread through the Lymphatic System

MBC cells mainly metastasize through the circulation system, and they can spread through lymphatic or hematogenous routes. The first metastatic sites are often the lymph nodes close to tumor, and therefore the presence of lymph node metastasis correlates with more metastasis and poorer prognosis [82]. Targeting the lymph nodes via nanoparticles could be one method to stop the spread of MBC cells. Since cancer cells elicit an immune response, nanotechnology can take advantage of this process and nanoparticles can be bound to the leukocytes and then transferred to lymph nodes together with therapeutic agents [83]. One successful strategy is using carbohydrate (e.g., dextran)-coated iron-oxide nanoparticles which have an improved accumulation in lymph nodes, due to the enhanced uptake by leukocytes in circulation [84,85,86]. Furthermore, another study demonstrated a therapeutic effect of an iron-loaded nanoparticle-ferumoxytol, which is now approved by the FDA for treating iron deficiency, that showed promise against early BCs and lung cancer metastases in the liver and lungs [87]. Increased mRNA levels associated with pro-inflammatory Th1-type responses was found on macrophages exposed to ferumoxytol, and cancer cells co-incubated with ferumoxytol showed elevated caspase-3 activity. This result suggests that iron-loaded nanoparticles could protect the distant organs from metastatic seeds by modulating macrophage responsiveness.

Another strategy is using the ultra-small nanoparticles with higher permeability to the lymphatic systems. Although the primary tumor and sentinel lymph nodes could be removed by the surgery, it is hard to treat the tumor cells still residing in the lymphatic vessels [88,89]. The prevention of lymphatic metastasis is critical for clinical MBC treatment, and the role of the lymphatic system has been the under the direct attention for the development of suitable drug-delivery strategies. Ultra-small nanodrugs (10~30 nm in diameter) have been successfully applied against tumor metastasis, whose mechanisms can be directly related to their lymphatic accumulation [90,91,92,93].

### 6.2. Interfering with the Colonization of Distant Organ Sites

Metastatic suppressor genes provide gene targets for the regulation of the colonization of distant organ sites within the metastatic cascade. Promoting metastatic dormancy through metastatic suppressor genes has proven to be an effective way to prevent colonization of the already disseminated MBC cells. The first and most reported metastatic suppressor gene is the human nonmetastatic gene 23 (*Nm23-H1*), which could abrogate metastasis development without growth inhibition of the primary tumor [94]. Mechanistically, lysophosphatidic acid receptor 1 gene (*LPA1*) has an inverse expression and functional interaction with *Nm23-H1*, and the inhibitor of *LPA1* (Debio-0719) was found to effectively function as a metastatic suppressor, which could induce metastatic dormancy of MBC cells [95]. As a highly hydrophobic small molecule, Debio-0719 seems to have minimal chances being applied in real clinical activities, but it would be expected to take advantage of nanotechnology to solve the poor solubility in physiological aqueous environment. However, Debio-0719 presents a new drug development paradigm to induce MBC cell dormancy in metastatic sites.

Anti-apoptosis is an important mechanism of metastatic cancer cells [96], and therefore a critical intervention point for the treatment of metastasis. Recent studies have also demonstrated that the apoptotic resistance of CSCs is essential for metastasis, and recent studies have proved that the induction of apoptosis in these cells could prevent metastasis. Several lines of evidence support this hypothesis. First, it has been shown that the metastatic potential of both human and murine cells is positively correlated with the anti-apoptotic propensity of the cells when injected into immunocompromised mice [97]. Second, experimentally-manipulating key anti-apoptotic or pro-apoptotic factors could influence overall metastatic efficiency, as BC cells overexpressing exogenous BCL2 induced an increased number of lung metastases in vivo [98]. Third, the inactivation of pro-apoptotic genes promotes metastasis. Loss of *p53* function has been associated with MBC metastasis clinically [99]. Similarly, *HCCR-2*, human cervical cancer oncogene encoding a negative modulator of *p53*, could promote transgenic mice to develop BC and metastasis [100].

Moreover, data from human metastatic tumors indicate that the gain-of-function of anti-apoptotic genes (e.g., *survivin*, *Nuclear Factor κB (NFκB)* or *BCL2*) or the loss of function of pro-apoptotic genes (e.g., *BAX*, *p53* and *death-associated protein kinase (DAPK)*) closely correlate with the tumor progression [101,102]. Particularly, studies have focused on survivin because it was found that expression is low in normal tissues [103], and overexpressed in most malignant tumors [104]. It has also been reported that TAT-g-CS/siRNA nanoparticles targeting survivin strongly inhibited the proliferation of TNBC cells via inducing cell apoptosis both in vitro and in vivo, and thus reduced TNBC metastasis in vivo [9]. Conversely, DAPK is a positive apoptosis mediator and could be used for the suppression of in vivo metastasis [101]. Hence, we stress the induction of apoptosis in CSCs has a crucial role during the metastasis treatment, which could be taken advantage of by nanomedicine design.

## 7. Summary and Future Perspectives

This review summarizes the latest progression of NP-mediated drug/gene delivery systems against cancer within the past few decades, focusing on the advancement of using nanomedicine for MBC treatment. However, despite the great improvements of Abraxane^®^ in MBC treatment, substantial efforts are still needed to overcome the physiological barriers hindering nanoparticle delivery to metastases. Furthermore, we discussed nanomedicine designing strategies based on the biological development of MBC. To translate these applications into real clinical use, scientists must further optimize the nanomaterials to adapt to the challenges of a sophisticated in vivo environment. The summarized gene delivery systems in this review exhibit great potential against MBC, but still need extra effort to adapt to use in vivo. The use of targeting ligands specific for overexpressed receptors at metastatic sites, is still a driving force for the development of nanomedicines. Continued improvements of nanomedicines will lead to advancements of clinical testing for MBC patients apart from Abraxane^®^.

## Figures and Tables

**Table 1:** Status of various ncRNA-based nanomedicine under clinical trial.

Name	Agent	Delivery System	Indications	Company	Current Status	Identifier
EphA2 targeting DOPC-encapsulated siRNA	siRNA	LNP	Advanced cancers	MD Anderson Cancer Center	phase 1	NCT 01591356
Atu027	siRNA	LNP	Advanced solid tumors	Silence Therapeutics GmbH	phase 2	NCT 00938574
PRO-040201	siRNA	LNP	Hypercholesterolemia	Tekmira	phase 1	NCT 00927459
TKM-080301	siRNA	LNP	Multiple cancers	Tekmira	phase 1	NCT 01437007
ALN-VSP02	siRNA	LNP	Solid tumors	Alnylam	phase 1	NCT 01158079
TKM-100201	siRNA	LNP	Ebola-virus infection	Tekmira	phase 1	NCT 01518881
ALN-PCS02	siRNA	LNP	Elevated LDL-Cholesterol	Alnylam	phase 1	NCT 01437059
ALN-TTR02	siRNA	LNP	Amyloidosis	Alnylam	phase 3	NCT 02510261
DCR-MYC	siRNA	LNP	Hepatocellular Carcinoma	Dicerna	phase 2	NCT 02314052
MRX34	miRNA mimic	liposome	various solid tumor	Mirna Therapeutics	phase 1	NCT 01829971
TargomiRs	miRNA mimic	minicells	Malignant Pleural Mesothelioma Non-Small Cell Lung Cancer	Asbestos Diseases Research Foundation	phase 1	NCT 02369198
CALAA-01	siRNA	cyclodextrin polymer-based nanoparticle	Various solid tumors	Calando	Phase 2	NCT 00689065
siG12D LODER	siRNA	LODER polymer	Pancreatic cancer	Silenseed Ltd.	phase 2	NCT 01676259

DOPC: 1,2-dioleoyl-sn-glycero-3-phosphatidylcholine.

**Table 2:** Cancer-treating nanomedicines already approved or being applied in the clinical trials.

Brand	Delivery System	Indications	Company	Current Status
NanoTherm^®^	Iron oxide nanoparticle	Local treatment of glioblastomas	MagForce AG	Approved in Germany
Aurimmune^®^	Colloidal gold-bound recombinant human tumor necrosis factor	Pancreatic cancer	CytImmune Sciences	Phase 2 clinical trial
Doxil^®^(US) [Caelyx^®^(Europe)]	PEGylated liposome doxorubicin	Ovarian/BC	Orthobiotech, Schering-Plough	FDA approved
Abraxane^®^	Albumin-bound Paclitaxel nanoparticles	Various cancer therapy	Abraxis Bioscience	FDA approved
	Nab paclitaxel in combination with gemcitabine	Metastatic pancreatic cancer	Celgene	FDA approved
Myocet^®^	Non-PEGylated liposome of Doxorubicin	BC therapy	Elan Pharmaceuticals/Sopherion Therapeutics	Approved in Europe and Canada
DaunoXome^®^	Liposome-encapsulated Daunorubicin	Advanced HIV-associated Kaposi sarcoma	Gilead Science	FDA approved
DepoCyt^®^	Liposomal Cytarabine	Lymphomatous meningitis	Pacira Pharms Inc.	FDA approved
Oncaspar^®^	PEGylated L-asparaginase	Acute Lymphocytic Leukemia	Sigma Tau	FDA approved
Onco-TCS^®^	Liposomal Vincristine	Non-Hodgkin Lymphoma	Inex	Phase 1/2 clinical trial
LEP-ETU^®^	Liposomal Paclitaxel	Ovarian/breast/lung cancers	Neopharma	Phase 1/2 clinical trial
Aroplatin^®^	Liposomal Cisplatin analog	Colorectal cancer	Antigenics, Inc.	Phase 1/2 clinical trial
OSI-211	Liposomal Lurtotecan	Lung cancer/recurrent ovarian cancer	OSI	Phase 2 clinical trial
SPI-77	PEGylated liposomal Cisplatin	Head and Neck cancer/Lung cancer	Alza	Phase 3 clinical trial
EndoTAG-1	Paclitaxel embedded in liposomal membranes	BC/Pancreatic cancer	Medigene/SynCore Biotechnology	Phase 2 clinical trial
Marqibo^®^	Vincristine	Philadelphia chromosome-negative lymphoblastic leukemia	Talon Therapeutics	FDA approved
ThemoDox^®^	Doxorubicin	Hepatocellular carcinoma	Celsion Corporation	Phase 3 clinical trial
Atragen^®^	Liposomal all trans-retinoic acid	Acute promyelocytic leukemia	Aronex Pharmaceuticals	Phase 2 clinical trial
Lipoplatin^®^	Liposomal Cisplatin	Pancreatic/Head and Neck/BC	Regulon	Phase 3 clinical trial
Aurimmune^®^ (CYT-6091)	TNF-α bound to colloidal gold nanoparticles	Head and Neck cancer	Cytimmune Sciences	Phase 2 clinical trial
Auroshell^®^	Gold nanoshell	Thermally destroy the tumor tissue	Nanospectra Bioscience	Phase 1 clinical trial
Genexol-PM^®^	Polymeric micelle loaded with paclitaxel	BC/small cell lung cancer	Samyang	Approved in Europe and Korea
Paclical^®^	Paclitaxel micelles	Ovarian cancer	Oasmia Pharmaceutical AB	Phase 3 clinical trial
Narekt-102	PEGylated liposome loaded with Irinotecan	Breast/Colorectal cancer	Nektar Therapeutics	Phase 3 clinical trial
NKTR-105	PEG-Docetaxel conjugate	Solid tumors	Nektar Therapeutics	Phase 1 clinical trial
